# Unified model of short- and long-term HIV viral rebound for clinical trial planning

**DOI:** 10.1098/rsif.2020.1015

**Published:** 2021-04-14

**Authors:** Jessica M. Conway, Paige Meily, Jonathan Z. Li, Alan S. Perelson

**Affiliations:** ^1^ Department of Mathematics and Center for Infectious Disease Dynamics, Pennsylvania State University, University Park, PA, USA; ^2^ University of Pennsylvania School of Arts and Sciences, Philadephia, PA, USA; ^3^ Brigham and Women’s Hospital, Harvard Medical School, Boston, MA, USA; ^4^ Theoretical Biology and Biophysics, Los Alamos National Laboratory, Los Alamos, NM, USA

**Keywords:** HIV, viral rebound, latency, mathematical modelling

## Abstract

Antiretroviral therapy (ART) effectively controls HIV infection, suppressing HIV viral loads. Typically suspension of therapy is rapidly followed by rebound of viral loads to high, pre-therapy levels. Indeed, a recent study showed that approximately 90% of treatment interruption study participants show viral rebound within at most a few months of therapy suspension, but the remaining 10%, showed viral rebound some months, or years, after ART suspension. Some may even never rebound. We investigate and compare branching process models aimed at gaining insight into these viral dynamics. Specifically, we provide a theory that explains both short- and long-term viral rebounds, and post-treatment control, via a multitype branching process with time-inhomogeneous rates, validated with data from Li *et al.* (Li *et al.* 2016 *AIDS* **30**, 343–353. (doi:10.1097/QAD.0000000000000953)). We discuss the associated biological interpretation and implications of our best-fit model. To test the effectiveness of an experimental intervention in delaying or preventing rebound, the standard practice is to suspend therapy and monitor the study participants for rebound. We close with a discussion of an important application of our modelling in the design of such clinical trials.

## 1. Introduction

With the advent of antiretroviral therapy (ART), the prospects of people living with HIV (PLWH) improved immeasurably, with ART improving both the quality and length of life [1,2]. However ART is not a cure; while regular dosing with ART does effectively control the infection and hold the amount of circulating virus below the level detectable by clinical assays, suspension of therapy is typically followed by HIV rebound to high viral loads [3].

Recent results give significant nuance to ‘typical’ viral rebound following analytic treatment interruption (ATI). In a 2013 prospective study, a cohort of 14 PLWH (the VISCONTI cohort) were identified, who were able to control HIV infection for a prolonged period—upwards of a decade—after stopping ART [4]. Results from the VISCONTI study and others suggest that post-treatment controllers (PTCs) may control HIV using a mechanism distinct from that of spontaneous HIV controllers [5,6]. Li *et al.* identified a distinct cohort of PTCs, who maintained viral loads less than or equal to 400 HIV RNA copies ml^−1^ for greater than or equal to 24 weeks [7,8], in a pooled analysis of participants from AIDS Clinical Trials Group (ACTG) studies, the Montreal Primary HIV Infection cohort, the Seattle Primary Infection Program, and the Ragon HIV Controllers cohort. Since ART comes with a number of drawbacks including side-effects and cost, the search for biological indicators (biomarkers) of lasting ART-free HIV remission has become a priority in HIV cure research [9,10], with some good progress made [11–15]. Another significant priority is to find treatments and treatment strategies that may induce ART-free remission or even HIV cure [16,17]. Recently, two promising strategies were described, employing latency-reversing agents and immune system modulators [18,19].

When investigating the efficacy of interventions intending to delay or prevent rebound, the standard is stop therapy and observe whether rebound occurs via regular viral load tests. However, there is significant heterogeneity in HIV viral dynamics following ATI: in a pooled analysis of outcomes from six ACTG ATI studies, Li *et al.* reported widely varying times to viral rebound, with a significant number of participants maintaining viral suppression to undetectable levels for up to two or more months in the absence of ART [12]. A good characterization of such dynamics would be of significant use in demonstrating efficacy of interventions that may delay rebound.

An obvious toolset would be survival analysis, which is used to analyse the expected duration of time until one or more events—in this case, duration until viral rebound. However viral rebound dynamics are not well predicted by standard stylized survival distributions, e.g. the Weibull or the lognormal distribution, as we show in the Results (§3.1, figure 2). More specifically, they reasonably predict short-term viral rebounds, within a few months, but fail to describe delayed viral rebound that occurs after many months or years. To make up for this deficit, we derive a mechanistically motivated survival function that more accurately describes both short- and long-term viral rebound.

Within-host modelling of HIV infection is a well-established field [20–29]. Much of our quantitative understanding of HIV dynamics within a host, e.g. the infected cell death rate, the rate of viral clearance, and the number of progeny virions released from an infected cell, i.e. the burst size, derive from models fit to data [30–32]. Existing models have mainly focused on the kinetics of early infection and the effects of treatment. But recent years have seen the development of models of HIV post-treatment control [28] and the time to viral rebound after treatment cessation. Hill *et al.* [33,34] provided estimates of viral rebound time distributions, used in combination with careful and thoughtful consideration of within-host parameters, to evaluate the needed efficacy of therapeutic agents that might one day yield HIV cure. First estimates of HIV recrudescence rates were provided by Pinkevych *et al.* [35,36]. Fennessey *et al.* investigated SIV viral rebound in macaques infected with barcoded virus, to generate more detailed insights into viral rebound [37]. Building on these insights, we integrated biomarkers, specifically an individual’s levels of cell-associated HIV RNA (HIV CA-RNA) and ART regimen pre-analytic treatment interruption (ATI) [12,38], into a stochastic model to start to move towards personalized viral rebound time predictions [39]. Working towards more personalization, Bing *et al.* [40] recently demonstrated that both empirical and mechanistic modelling approaches with post-ATI viral load data identify the same predictors of viral rebound, giving modelling support to identification of rebound delay predictors including pre-ATI ART regimen [12,38,39], time of initiation of ART [12] and higher nadir CD4+ T-cell counts [40]. And in a different vein, Wu *et al.* used the barcoded virus data from [37] to investigate the impact of hypothesized fluctuations in activation rates over time [41]. Notably, these models have mainly emphasized viral rebound over short timescales, in part because rebound datasets are small and therefore seldom include data on rare long-term viral rebounds. In this present study, we use data from Li *et al.*’s pooled analysis of six ATI studies [12], with 235 total study participants, large enough to begin to characterize both short-term rebound and long-term rebound simultaneously.

As with previous models, we rely primarily on the hypothesis that viral rebound is driven by latent cell activation [28,33–36,39,40]. However, the latent reservoir is heterogeneous, with recent studies suggesting it is composed of clones with different antigen specificity [42,43] and different integration sites [44], factors that can influence the propensity of latently infected cells to reactivate and produce replication competent virus. Further there are indications that immune responses play a role in determining rebound times, with studies suggesting that significantly delayed rebounds may be associated with additional mechanisms of infection control, such as anti-HIV immune responses [28], and T-cell exhaustion markers are predictive of a shorter time to viral rebound [15]. To model the impact that this heterogeneity may have on latent cell activation inducing viral rebound, we will investigate models of latent cell activation that are heterogeneous in time, which serves as a contrast to most previous studies [35,36,39,40,45] which assumed constant latent cell activation rates, with Wu *et al.* [41] being an exception. Thus, we aim to capture both short- and long-term viral rebound.

In the following, we will investigate models of viral rebound that offer improved survival distributions, i.e. a cumulative probability density function for the probability of an individual’s viral rebound at time *t*. Armed with an effective simple model we will discuss the biological insight offered by parameter estimates obtained by fitting the model to data. In particular, we show that one can derive from our model the number of patients in an ATI clinical trial needed to detect an intervention-associated viral rebound delay relative to baseline.

## 2. Methods

### 2.1. Description of data

The description of the data we employ and associated collection methodologies are fully explained in [12]. Briefly, participants in six ACTG ATI studies (ACTG 371 [46], A5024 [47], A5068 [48], A5170 [49], A5187 [50] and A5197 [51]) were included if they were on suppressive ART, received no immunologic interventions (e.g. therapeutic vaccination, interleukin-2), and had HIV-1 RNA less than 50 copies ml^−1^ at the time of ATI (*N* = 235 participants). Early after treatment interruption, most studies reported weekly viral load measurements, with the exception of A5170 for which the median interval between viral load measurements was 5 days within six weeks ATI, though the interquartile range is broad, from 2 to 20 days. Viral rebound was defined as sustained viral loads of at least 200 HIV RNA copies ml^−1^.

In a previous study [39], we restricted our analysis to the subset of participants that had peripheral blood mononuclear cells (PBMCs) and plasma available for HIV reservoir quantification while on ART prior to the ATI, that had CA-RNA above the level of detection at ATI, and who showed viral rebound less than or equal to 60 days after ART cessation (*N* = 84 participants). However, here we aim to develop a mechanistically motivated, phenomenological model that describes both short- and long-term viral rebound in all patients that undergo ATI and analyse data from all *N* = 235 study participants.

Finally, we acknowledge that defining viral rebound is challenging when examining data from real study participants. We define viral rebound as having occurred following a first viral load measurement above 50 copies ml^−1^ given all subsequent viral load tests show detectable viraemia, ideally above 200 copies ml^−1^. Some judgement was applied in cases where those conditions were not met (e.g. study participant 14 594, see electronic supplementary material figure S2). Viral dynamics for all study participants under consideration are provided in electronic supplementary material figure S2, with the last undetectable/first detectable viral load measurements we employ for our parameter estimation marked in blue and red, respectively.

### 2.2. Model

**Figure 1.**
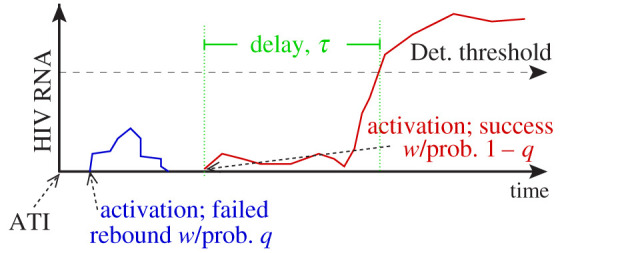
Model schematic [39]. Following ATI, we assume that latent cell activations are followed by chains of infection that successfully re-establish high viral loads associated with chronic infection, with probability 1 − *q*, or die out, with probability *q*. We assume further a delay *τ* between a successful activation and the time when plasma viraemia is detectable.

Our baseline model was described in [39]. In brief, as in previous studies we assume that activation of latently infected cells drives viral rebound [28,34,35,39]. We envision ensuing dynamics as illustrated in figure 1. We do not assume that all latently infected cell activations cause viral rebound. Rather, we assume that activation is followed by rounds of viral replication, which may cause viral populations to grow to detectable levels, thereby causing viral rebound, or to die out. We define *q* as the probability of ‘die out’, i.e. that the activation of a latently infected cell does not cause viral rebound. Then 1 − *q* is the probability of a ‘successful latent cell activation’, which does cause viral rebound. Finally, we assume that there is a delay between successful latent cell activation and detectable infection, where ‘detectable infection’ corresponds to the study participant’s viral loads exceeding 50 HIV RNA copies ml^−1^. For simplicity, we take a fixed, delta-distributed delay; in a previous study [39], we found that, while a Weibull distribution best described the delay, per the Akaike information criterion (AIC), the improvement over other stylized distributions, including a fixed delay, was not statistically significant [39].

Taking the latent reservoir size at the time of ATI and later to be *L*_0_, and assuming that the latently infected cells are activated at *per capita* rate *a*, infected cell influx occurs at the constant rate *aL*_0_. Then the recrudescence (successful activation) rate is *r* = (1 − *q*)*aL*_0_. Because the latent reservoir has a half-life of approximately 44 months [52,53], and our dataset extends only about 2 years post-ATI, assuming a constant latent reservoir may introduce little error. Nonetheless, later we relax this assumption.

From a mathematical standpoint, we employ a branching process framework to construct from these assumptions a probability of viral rebound at time *t*. Given the recrudescence rate *r* = *aL*_0_(1 − *q*), from a simple branching (immigration) process formulation we can compute the cumulative probability of successful activation at time *t*, *P*_ACT_(*t*) = 1 − exp ( − (1 − *q*)*aL*_0_*t*) (see [39] for derivation). Then, assuming a fixed delay *τ*, i.e. delta-distributed delay *δ*(*t* − *τ*), the probability of viral rebound by time *t* predicted by our model is

2.1 $$P_{\mathrm{VR}}(t)=\left\{ \begin{array}{ll} 0,\;\; & 0\leq t<\tau \\ 1-e^{-(1-q)aL_{0}(t-\tau )}, & t\geq \tau \;\; \end{array}\right.$$

(see again [39]). We call this is the baseline model; below we will also investigate alternate, time-inhomogeneous, formulations for the recrudescence rates and derive the associated *P*_VR_(*t*). We will use these to derive likelihood functions,

2.2 $$\mathrm{lik}(\theta )=\prod_{i=1}^{235}(P_{\mathrm{VR}}(t_{\mathrm{first}}^{\left(i\right)};\theta )-P_{\mathrm{VR}}(t_{\mathrm{last}}^{\left(i\right)};\theta )),$$

where *P*_VR_(*t*) is the cumulative probability of viral rebound, multiplied over the 235 study participants, ***θ*** are the parameters, and $t_{\mathrm{first}}^{\left(i\right)}$ and $t_{\mathrm{last}}^{\left(i\right)}$ are the times of the first detectable and last undetectable viral load measurements, respectively, for the *i*th study participant. Note that in using this likelihood function equation (2.2), we do not estimate viral rebound times from the data, e.g. by the mid-time point between the last undetectable and first detectable viral load measurement, in advance of the analysis. We therefore avoid the subtle uncertainty such pre-processing can create, particularly as we aim to use data that includes study participants with viral load measurements that are months apart. For all models, we estimate parameters using maximum-likelihood methods, and compare model fits using the AIC [54]. Thus, we test how well the models explain observations of viral rebound [12].

## 3. Results

We begin by providing a motivating result, showing that viral rebound dynamics are not well predicted by standard stylized survival distributions, e.g. the Weibull or the lognormal distribution, as alluded to in the Introduction (§1). This observation provided the impetus for us to derive our mechanistically based viral rebound time distribution.

We document our first effort, estimating model parameters for the baseline model, equation (2.1), and show that this simple model does not improve predictions of long-term viral rebound relative to the standard survival distributions. We then discuss and justify alternate hypotheses for recrudescence, showing that a recrudescence rate which decreases with time explains the data much better. Finally, we discuss how our model predictions can be used to inform clinical trial design.

### 3.1. Viral rebound dynamics are not well predicted by standard stylized survival distributions

**Figure 2.**
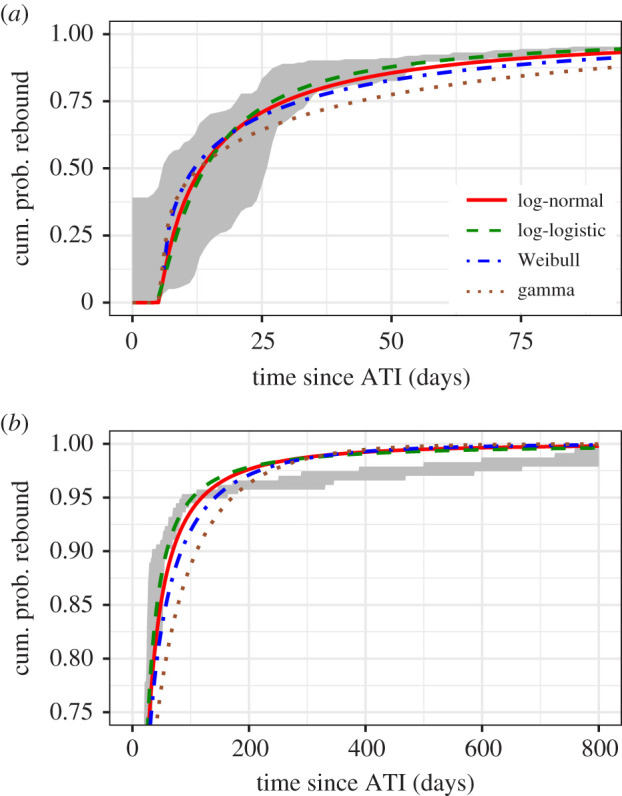
Typical survival curve distributions fit to viral rebound data [12], shown over (*a*) short timescales and (*b*) long timescales. The shaded region bounds the empirical distributions for the times of last undetectable and first detectable viral load tests across all study participants. To fit the distribution parameters, we define a likelihood $\mathrm{lik}(\theta )=\prod_{i=1}^{235}(P_{\mathrm{VR}}(t_{\mathrm{first}}^{\left(i\right)};\theta )-P_{\mathrm{VR}}(t_{\mathrm{last}}^{\left(i\right)};\theta ))$ where *P*_VR_(*t*) is the cumulative distribution (log-normal, log-logistic or Weibull), with parameters ***θ***, and $t_{\mathrm{last}}^{\left(i\right)}$ and $t_{\mathrm{first}}^{\left(i\right)}$ correspond to the last undetectable and first detectable viral load, respectively, for study participant *i*, summed over 235 study participants for whom we have data [12]. To maximize the likelihood, we employed the Davidon–Fletcher–Powell optimization algorithm implemented by the Bhat package in R [55]. The data are described in §2.1.

Survival analysis provides a natural toolset to analyse time-to-event data such as the HIV viral rebound data that forms the focus of this present study. However, when fitting the data to standard stylized survival distributions, i.e. Weibull, lognormal, log-logistic or gamma distributions, we find that viral rebound dynamics are not well predicted by these models. While the log-logistic distribution gives the best fit per the AIC (see electronic supplementary material, table S1), visual inspection of the fits in figure 2 suggests that while they reasonably predict short-term rebound, within a few months of ATI, they fail to describe delayed viral rebounds occurring after many months or years. To estimate parameters for the survival distributions, we use the likelihood function in equation (2.2) with the cumulative density function of the desired distribution as *P*_VR_. To maximize the likelihood, we employed the Davidon–Fletcher–Powell optimization algorithm implemented by the Bhat package in R [55].

What follows is a derivation and justification of our mechanistically motivated survival function that more accurately describes both short- and long-term viral rebound to make up for the deficit presented by stylized distributions. Since the log-logistic distribution explains the data best, in the following it will be used as a comparator to quantify the improvements that our model brings.

### 3.2. Constant *r* fails to explain both short- and long-term rebound

**Figure 3.**
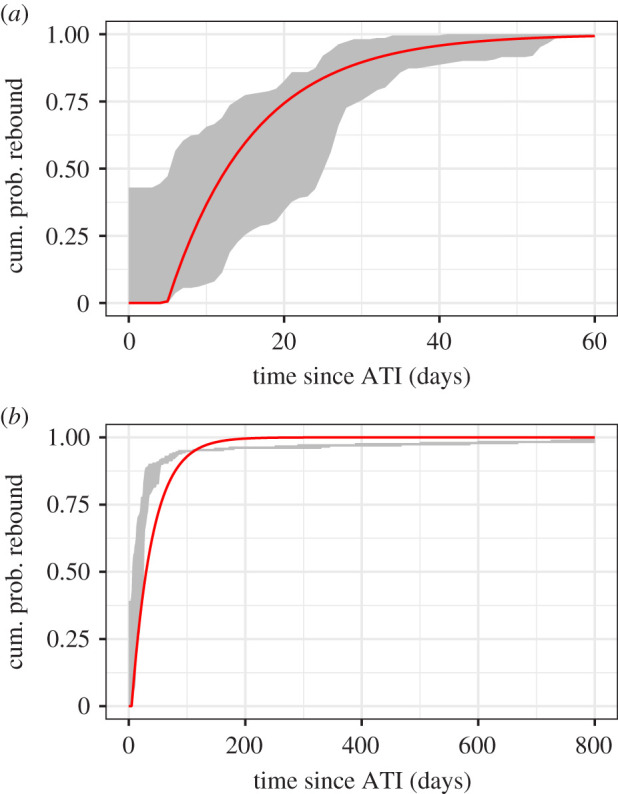
Baseline, constant recrudescence rate model, equation (2.1), fit to viral rebound data (see §2.1), with parameters estimates for data on (*a*) short timescales, less than or equal to 60 days only, and (*b*) short and long timescales, up to 800 days of observations. The shaded region bounds the empirical distributions for the times of last undetectable and first detectable viral load tests across all study participants.

We start by fitting the baseline model, equation (2.1), to the data, using maximum likelihood methods. Specifically we use the Davidon–Fletcher–Powell optimization algorithm to estimate model parameters, the recrudescence rate *r* = *a*(1 − *q*)*L*_0_ and the delay *τ*. Note that we cannot fit *a* or *q* individually because, with the infectious latent reservoir size *L*_0_, they form a non-identifiable parameter combination. A summary of these parameter estimates for the baseline model are provided in table 1 and the best fit of the model to the data is shown in figure 3. Note that we found that the data for long times was too sparse to characterize a delay, so to estimate the delay *τ* we used an estimate derived by fitting the model only to rebounds within 60 days.

**Table 1.** Parameter estimates for the baseline model equation (2.1), assuming a constant recrudescence rate *r*. ΔAIC is computed relative to the best stylized distribution, the log-logistic, see electronic supplementary material, table S1. Note that when estimating the recrudescence rate from all the data, we used the delay *τ* estimated using data restricted to rebounds less than 60 days from ATI.

time window	parameters	estimates (95% CI)	ΔAIC
rebound within 60 days of ATI	*r* = (1 − *q*)*aL*_0_	0.0902 (0.0771,0.1054) per day	n.a. (restricted data)
	delay *τ*	4.93 (4.24,5.70) days	
rebound across all observed times	*r* = (1 − *q*)*aL*_0_	0.0278 (0.0231,0.0335) per day	252.6 (with one parameter)
	delay *τ*	4.93 days fixed

Assuming a constant recrudescence rate explains short-term viral rebound data reasonably well (figure 3*a*). However, even a visual inspection shows that the model only poorly explains both short- and long-term viral rebound data simultaneously (figure 3*b*). This is borne out in comparisons of AIC, which shows poor performance relative to most survival distributions (i.e. it increases the AIC such that ΔAIC > 250 for best-fit, log-logistic distribution), though it does offer an improvement on the worst-fit gamma-distribution assumption (ΔAIC ≈ 4) (electronic supplementary material, table S1). Thus while our model assuming a constant recrudescence rate does explain short-term viral rebound (see also [39]) it does not explain both short- and long-term viral rebound, nor does it offer any improvement over standard survival analysis distributions.

### 3.3. Exponentially decaying *a*(*t*) explains both short- and long-term viral rebound

The failure of our baseline model, which assumes a constant recrudescence rate, is not really a surprise. For one, it relies on the assumption of a constant replication-competent latent reservoir size, and studies have shown that the latent reservoir size usually decays over time in the presence of effective therapy [52,53]. Presumably, the reservoir would similarly continue to decay post-ATI but pre-rebound, since the viral load remaining undetectable implies that robust viral replication, which may replenish the reservoir, is unlikely. Further, we recently showed that short-term viral rebounds are actually better explained by multiple successful latent cell activations in succession, with detectable viraemia composed of virus arising from replication in multiple lineages [56]. Lastly and importantly, we completely neglected the inherent heterogeneity of the latent reservoir. Post-ATI viral rebound can occur in PLWH with markers indicating small viral reservoirs [12,57,58], suggesting reservoir characteristics beyond size are associated with rebound. The latent reservoir is composed primarily of memory cells [59], each of which may be specific for a pathogen or set of pathogens. Recent characterization suggests that the reservoir is largely made up of clonal populations [60–62], with different antigen specificities [42,43], so there may be genetically homogeneous subsets of cells. While in principle, we can neglect this heterogeneity in favour of a mean activation rate given that the latent reservoir is large in most PLWH, perhaps depletion of clones with higher activation rates, e.g. those that recognize common antigen, changes the average activation time in a manner that is significant over longer timescales.

These observations motivate investigation of time-inhomogeneous recrudescence rates that decay in time. The modelling attempt in the previous section supports this choice: in comparing figure 3*a*,*b*, we note that the recrudescence rate decreased in order to accommodate long-term viral rebounds. For simplicity, we investigate exponentially decaying recrudescence rates, both single- and biphasic decay. We are guided in our choice by the exponential decay, on average, of the latent reservoir size [52,53]. As the time-dependent recrudescence rate *r*(*t*) = (1 − *q*(*t*))*a*(*t*)*L*(*t*), the source of the time-inhomogeneity may be in the activation rate, the probability that a latent cell activation leads to rebound, and the latent reservoir size. Thus, more generally, the probability of viral rebound is given by

3.1 $$P_{\mathrm{VR}}(t)=\left\{ \begin{array}{ll} 0, & 0\leq t<\tau \\ 1-\exp\left(-\int_{\tau }^{t}r(s)\;ds\right), & t\geq \tau . \end{array}\right.$$

**Figure 4.**
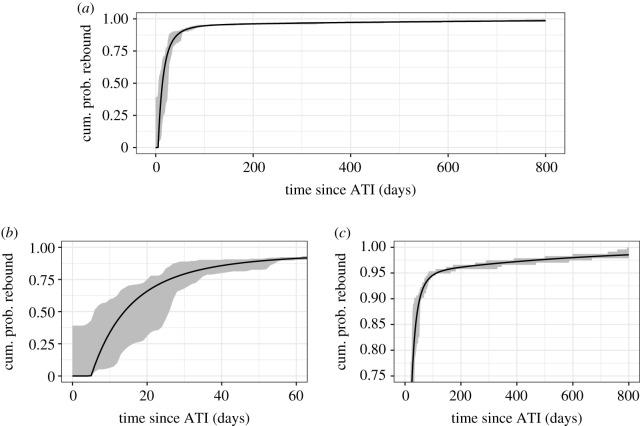
Cumulative probability of viral rebound assuming a single-phase exponentially decaying recrudescence rate model (model (2), see table 2), (*a*) over all time since ATI, (*b*) horizontally zoomed in to early times, to show quality of fit to early-rebound data, and (*c*) vertically zoomed in to highlight the quality of the fit to late-rebound data, not captured by the standard survival distributions tested (figure 2) The shaded region bounds the empirical distributions for the times of last undetectable and first detectable viral load tests across all study participants.

The results of fitting this model to the data are summarized in table 2. First, across all models, the delay *τ* is remarkably consistent, on the order of 4–5 days (cf. tables 1 and 2, and electronic supplementary material table S1). Further, the data are best explained by the recrudescence rate model (2) in table 2, where the recrudescence rate decays exponentially over time to a constant, non-zero rate. Figure 4 shows that recrudescence rate model (2) effectively recapitulates both the short- and long-term viral dynamics simultaneously not only better than previous models, but very well. We will use this model in our discussion of clinical trial design that follows.

**Table 2.** Parameter estimates for viral rebound models assuming different time-dependent recrudescence rates. ΔAIC is computed relative to the best-fit stylized distribution, the log-logistic, with parameter estimates in electronic supplementary material, table S1.

	recrudescence rate (*r*(*t*)) model	parameters	estimate (95% CI)	ΔAIC
(1)	*r*(*t*) = *r*_0_ e^−*k*(*t*−*τ*)^	*r*_0_	0.058 (0.049,0.069) per day	77.7
	single-phase decay, with	*k*	0.011 (0.009,0.014) per day	
	*r*(*t*) → 0 as *t* → ∞	*τ*	4.04 (3.28,4.94) days	
(2)	*r*(*t*) = *r*_∞_ + (*r*_0_ − *r*_∞_)e^−*k*(*t*−*τ*)^	*r*_0_	0.088 (0.073,0.106) per day	−10.7
	single-phase decay, with	*r*_∞_	0.002 (0.001, 0.004) per day	
	*r*(*t*) → *r*_∞_ ≠ 0 as *t* → ∞	*k*	0.029 (0.022,0.040) per day	
		*τ*	4.98 (4.58,5.41) days	
(3)	$r(t)=r_{\infty }\;e^{-k_{2}(t-\tau )}+(r_{0}-r_{\infty })\;e^{-k_{1}(t-\tau )}$,	*r*_0_	0.088 (0.068,0.113) per day	−8.7
	where *k*_2_ < *k*_1_	*r*_∞_	0.002 (0.001,0.004) per day	
	biphasic decay, with	*k*_1_	0.029 (0.019,0.053) per day	
	*r*(*t*) → 0 as *t* → ∞	*k*_2_	5 × e^−07^ (1 × e^−10^, 0.002) per day	
		*τ*	4.98 (4.57,5.42) days

However, note that the estimated delay *τ*, the initial recrudescence rate *r*_0_, and the initial decay *k* are the same for recrudescence models (2) and (3), and that the increased ΔAIC of model (3) relative to model (2) results from the difference in the number of parameters (table 2). Moreover, there is significant uncertainty—over many orders of magnitude—in the second phase decay rate. A biphasic recrudescence rate model that decays to a constant (not shown) shows the same trends. From these observations, we conclude that while the recrudescence rate model (2), with uniphasic decay to a constant rate, is the most parsimonious model with the data we have, a repeat of this analysis with more data on post-ATI rebound times may yield a best model with a multi-phasic recrudescence decay rate.

### 3.4. Application: clinical trial design

The improvement in how well our single-phase exponentially decaying recrudescence rate model explains the data relative to standard survival analysis distributions as well as the constant recrudescence rate model, and goodness of fit, are clear (see figure 4 and tables 1 and 2). We therefore envision an important application of our results to be ATI clinical trial design. Our model provides an analytic form and parameter estimates for the time to viral rebound in the absence of any intervention. Here we offer a key example application, predicting the number of study participants required to detect a viral rebound delay with some desired statistical power.

The role the recrudescence rate plays in our model of viral rebound is that of the hazard function, i.e. the rate that rebound happens at time *t*, given that it has not yet occurred. Thus, we have shown that the hazard rate

$$h(t)\equiv r(t)=r_{\infty }+(r_{0}-r_{\infty })\;e^{-k(t-\tau )}$$

best explains the current data (table 2). Incidentally, this is why the Gompertz–Makeham distribution [63] was excluded among our stylized distributions (figure 2): its associated hazard rate has the same functional form but is exponentially increasing, rather than decreasing.

**Figure 5.**
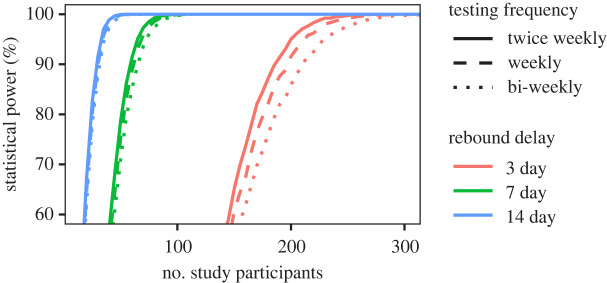
Statistical power of a study to detect a therapy-induced viral rebound delay depending on the number of trial participants, assuming a 3-, 7- or 14-day delay in median time to viral rebound, computed over 10 000 simulated clinical trials lasting 60 days (curves in red, green and blue, respectively), see electronic supplementary material, section A.2 for calculation details. Here we focus on detecting the difference in median rebound time. The line type indicates the assumed testing frequency: twice weekly (solid), weekly (dashed), bi-weekly (dotted). The delay here is generated by assuming that there is an intervention that reduces the constant hazard ratio (HR) from HR = 1 to HR ≈ 0.8 (3 day delay); HR ≈ 0.6 (1 week delay) and HR ≈ 0.4 (2 week delay).

Figure 5 illustrates the type of useful predictions that we envision with our model. It shows the statistical significance of a 60-day study depending on the number of ATI study participants required to detect an increased delay following ATI, computed over 10 000 *in silico* trials, see electronic supplementary material, section A.2 for calculation details. For the purposes of this illustration, we assumed an equal number of participants in the intervention and placebo arms, but in general that need not be the case. We predict, for example, that to detect an intervention-derived 3-day delay in viral rebound with 90% statistical power, our model predicts that would require 210 study participants if testing was performed every two weeks. With a higher frequency of testing, say twice weekly, one would requires fewer study participants—186—to achieve the same statistical power. The number of study participants required to achieve the same power is also decreased if the intervention has greater effect: our model predicts that one would require 59 or 65 study participants, given twice-weekly or bi-weekly testing, respectively, to detect an intervention-induced delay of 7 days with 90% statistical power. For these estimates, we use our time-dependent recrudescence model, which explains the data and therefore provide the most reliable results. And even over a 60-day simulated trial, the more approximate models, e.g. the constant recrudescence model fit only to viral rebound occurring on or before day 60 (figure 3*a*), or the best-fit distribution from survival analysis, the log-logistic (figure 2), yield noticeable under- or overestimates, respectively, for study participants required to achieve some statistical power (see electronic supplementary material, figure S1). Thus, the accuracy provided by our refined model can provide a meaningful correction to simpler models used in clinical trial design.

Note that the above calculation focuses on detecting if rebound time samples are drawn from different distributions, and not on resolving the rebound-time distributions; for that, the number of required study participants would be higher. For this illustration, to create a delay *in silico* we effectively decreased the hazard ratio (HR),

$$\mathrm{HR}=\frac{\mathrm{hazard}\;\mathrm{with}\;\mathrm{intervention}}{\mathrm{hazard}\;\mathrm{without}\;\mathrm{intervention}}.$$

That is, we set the hazard with intervention, $\tilde{h}(t)$, to be the scalar multiple HR of our estimated hazard function *h*(*t*), i.e. $\tilde{h}(t)=\mathrm{HR}\;h(t)$. We then used a nonlinear solver to compute HR so that the median time to rebound associated with $\tilde{h}(t)$ is delayed by 3, 7 and 14 days relative to the median time to rebound associated with *h*(*t*). Thus, a constant HR of approximately 0.8, 0.6 and 0.4, yield median 3, 7 and 14 day delays, respectively. However, with good support for the biological hypotheses underlying our modelling, we can make similar predictions for interventions that target specific rebound mechanisms. For example, broadly neutralizing antibody interventions that improve immune control [64] would reduce *q*, the probability that latent cell activation induces rebound (cf. figure 1; recall that the recrudescence rate *r*(*t*) = *a*(*t*)*L*(*t*)(1 − *q*(*t*))).

## 4. Discussion

In this study, we developed a model that captures the distribution of times to both short- and long-term HIV viral rebound after therapy interruption and demonstrated its applicability to clinical trial design.

Our model predictions improve significantly upon standard survival models (cf. figures 2 and 4) by considering the underlying biology. Specifically, we rely on the common assumption that HIV viral rebound following treatment suspension is triggered by activation of latently infected cells [28,33–35,39]. While the assumption that recrudescence resulting from such activation occurs at a constant rate explains observations of short-term viral rebound (less than or equal to 60 days) [35,39], it does not explain long-term observations (figure 3). This failure is likely due to the high level of heterogeneity in the latent reservoir. This heterogeneity comes from a variety of sources. The cells in the reservoir each carry a HIV provirus that potentially has a unique ability to reactivate due to a combination of factors including its genetic sequence, the effects of its integration site in the host cell genome [65] as well as epigenetic modifications and the host cell phenotype, e.g. whether it is a central memory, effector memory or transitional memory cell [66]. Each of these cell subtypes have distinct transcriptional and epigenetic programmes that could influence the activity of the integrated HIV promoter. Additionally, biological noise and stochastic fluctuations in transcriptional activity could play an important role in the reversal of latency [67,68]. Lastly, a large fraction of the reservoir is made up of T-cell clones with different antigen specificity [42,43], leading to the possibility that clones with specificity for more common antigens get activated more frequently than clones with specificity for rare antigens. Thus, to the extent that antigen drives proliferation and activation of cells in the reservoir, the reservoir composition could evolve from containing a majority of memory cells to common antigens to a reservoir containing a majority of cells with specificity for rare infrequently encountered antigens [69]. Also, because some are in a deeper state of latency than other cells, possibly due to their integration site [65], the analogous argument could be made that cells in a less deep state of latency activate first successively leaving cells with deeper and deeper latency in the reservoir. Either of these scenarios would be consistent with our finding that the recrudescent rate declines with time, and biologically would be due to a combination of a decline in the per cell activation rate *a*(*t*) and in the size of the reservoir *L*(*t*). We found that a single-phase decay to a low, constant viral load best explained the data (figure 4 and table 2, model (2)). However, the decay rate was more than an order of magnitude larger than the observed rate of decline of the latent reservoir [52,53] (table 2), consistent with the notion that the activation rate is also decreasing.

We predict an initial average recrudescence frequency of once every 9.4–13.7 days (95% CI for 1/*r*_0_), which is less frequent than predictions from Pinkevych *et al.* [35]. But this result is not necessarily inconsistent: the difference is only a few days, and our prediction reflects an average over all study participants regardless of drug regimen. Li *et al.* showed in this same cohort a statistically significant delay in rebound in study participants whose ART regimen included NNRTIs (99/235 individuals), which we plan to investigate in a separate study. After several months, the frequency slows down to once every approximately 260–1400 days (approx. 95% CI for 1/*r*_∞_). Thus, our modelling suggests that we can roughly consider the latent reservoir as composed of two major populations: cells that activate frequently and deplete rapidly, and cells that activate infrequently. The latter cell population may also include cells in which proviral sequences were integrated further away from transcriptional start sites, thereby producing less virus on average following activation, or possess other structural correlates of viral control [44].

The uncertainty in the late-stage recrudescence rate or frequency reflects the paucity of long-term rebounders in this dataset (cf. table 2). Indeed, we showed that models with more refined decay profiles, e.g. biphasic, yielded the same likelihood as our best-fit uniphasic model, and were judged worse only because the additional parameters did not provide a better description of the data (cf. table 2). Further, our model, as well as others, assumes that the source of rebound is the latent reservoir [28,33–36,39], which in the presence of ART shows an average, slow decay [52,53]. Following ATI but before viral rebound, it is reasonable to say that the reservoir continues to decay since re-seeding can only be very limited [29], and therefore over long enough times the reservoir should be eliminated. Thus, ultimately we would expect the recrudescence rate to drop to zero. Therefore we anticipate, with the availability of more data, that a more refined decay profile may best explain the data, potentially reflecting sequential depletion of clonal populations of latently infected cells [42,43].

As noted above with regards to drug regimen, in this study we neglected potentially important covariates. Li *et al.* noticed that time of treatment relative to exposure, and pre-ATI ART regimen, both had statistically significant impact on time-to-rebound [12]. With regards to the ART regimen, inclusion of non-nucleoside reverse transcriptase inhibitors (NNRTIs) was associated with longer delays. Also, unsurprisingly, treatment during acute HIV was associated with longer rebound delays than treatment during chronic infection, for which one explanation is a necessarily smaller reservoir [12]. But intriguingly, early treatment initiation—defined in [12] as within six months of exposure to HIV—was associated with still longer rebound delays, pointing to adaptive immune responses as an important actor too. A model accounting for these covariates may yield better prediction and useful biological insights.

We anticipate that the most significant contribution of this study is to ATI clinical trial design since our model offers a marked improvement over survival and other models in terms of explaining the observed distribution of times to viral rebound. Different therapeutic strategies to extend or prevent viral rebound are under investigation (e.g. at the time of this writing, clinical trials include [70–77]). By uniting short- and long-term viral rebound into a single model, well-validated by data (figure 4), we provide an analytic form and parameter estimates for the time to viral rebound in the absence of any intervention. We demonstrated the utility of our model in a power analysis to predict the number of study participants required to detect short intervention-associated delays in viral rebound relative to baseline, depending on the planned testing frequency (figure 5). Our model is mechanistically motivated and considers dynamics of viral rebound. Thus, we can make predictions for interventions that target specific aspects influencing viral rebound. For example, in our recrudescence rate *r*(*t*) = *a*(*t*)*L*(*t*)(1 − *q*(*t*)), *q* reflects the probability that latent cell activation leads to rebound, as opposed to the engendered viral lineage going extinct (figure 1). Thus, one could extend our analysis and directly model how antibody interventions improve immune control [64,78] and lead to reductions in *q*, yielding more realistic analyses.

## Data accessibility

The data underlying the results in this study are human; ethical and legal restrictions therefore apply. The data are available from the AIDS Clinical Trials Group (ACTG) based on their standard policies. Specifically, the data used in this analysis are publicly available by sending an email to the Statistical and Data Analysis Core of the ACTG (sdac.data@sdac.harvard.edu). See https://actgnetwork.org/clinical-trials/access-published-data for additional details.
